# Effect of Water Absorption and Stacking Sequences on the Tensile Properties and Damage Mechanisms of Hybrid Polyester/Glass/Jute Composites

**DOI:** 10.3390/polym16070925

**Published:** 2024-03-28

**Authors:** Rudá Aranha, Mario A. Albuquerque Filho, Cícero de L. Santos, Tony Herbert F. de Andrade, Viviane M. Fonseca, Jose Luis Valin Rivera, Marco A. dos Santos, Antonio G. B. de Lima, Wanderley F. de Amorim, Laura H. de Carvalho

**Affiliations:** 1 Escuela de Ingeniería Mecánica, Pontifícia Universidad Católica de Valparaíso, Valparaíso 2340025, Chile; 2 Post-Graduate Program in Materials Science and Engineering, Federal University of Campina Grande, Campina Grande 58429-900, Brazil; mario_alberto1910@hotmail.com (M.A.A.F.); heckerdecarvalho@yahoo.com.br (L.H.d.C.); 3 Mechanical Engineering Department, Federal University of Campina Grande, Campina Grande 58429-900, Brazil; cicero.santos@ufcg.edu.br (C.d.L.S.); santos.marco@ufcg.edu.br (M.A.d.S.); antonio.gilson@ufcg.edu.br (A.G.B.d.L.);; 4 Petroleum Engineering Department, Federal University of Campina Grande, Campina Grande 58429-900, Brazil; tonyhebert@uaepetro.ufcg.edu.br; 5 Textil Engineering Department, Federal University of Rio Grande do Norte, Natal 59078-970, Brazil; viviane.muniz@ufrn.br

**Keywords:** hybrid composites, jute fiber, VARTM, water sorption, hybrid effect, tensile properties, water absorption, stacking sequence, temperature effect

## Abstract

The aim of this work is to analyze the effect of water absorption on the mechanical properties and damage mechanisms of polyester/glass fiber/jute fiber hybrid composites obtained using the compression molding and vacuum-assisted resin transfer molding (VARTM) techniques with different stacking sequences. For this purpose, the mechanical behavior under tensile stress of the samples was evaluated before and after hygrothermal aging at different temperatures: TA, 50 °C, and 70 °C for a period of 696 h. The damage mechanism after the mechanical tests was evaluated using SEM analysis. The results showed a tendency for the mechanical properties of the composites to decrease with exposure to an aqueous ambient, regardless of the molding technique used to conform the composites. It was also observed that the stacking sequence had no significant influence on the dry composites. However, exposure to the aqueous ambient led to a reduction in mechanical properties, both for the molding technique and the stacking sequence. Damage such as delamination, fiber pull-out, fiber/matrix detachment, voids, and matrix removal were observed in the composites in the SEM analyses.

## 1. Introduction

Materials undergo degradation due to external influences, leading to changes in their properties over time. Exposure to aggressive environments or factors such as light, humidity, and heat tends to alter the mechanical, chemical, and physical properties of materials [1]. Specifically for composite materials, the change in properties can occur not only through the degradation of the material components but also through the degradation of the interface formed by these components, also referred to as the matrix/reinforcement interface [2].

To assess the degradation of composites under real-service conditions, accelerated or natural aging tests are conducted, inducing physical and/or chemical degradation. One method to comprehend the material’s behavior when exposed to humidity is through water sorption tests. In these tests, the material is immersed in an aqueous solution, sometimes with a controlled temperature, to expedite the degradation process.

Water absorption in composites is influenced by various factors, such as the chemical affinity and type of matrix, temperature, polarity, diffusivity, hydrogen bond formation, as well as the nature, volumetric fraction, orientation, porosity, and geometry of fibers or fabrics [3,4,5]. Another factor that has a significant influence on water absorption in composites is the voids present in the structure, which tend to increase water absorption. Water absorption, in turn, tends to compromise the mechanical properties of the material through complex mechanisms, including partial or total swelling of the matrix, plasticization of the matrix, material loss, and formation of cracks [6,7].

Matrix swelling can be detrimental to the fiber/matrix interface, promoting the delamination of fibers and matrix and thus reducing the mechanical properties of the material [5,8,9,10]. Similarly, plasticization can decrease the material’s mechanical properties and alter the glass transition temperature [11]. However, there are reports where plasticization increases the fracture toughness of the material, delaying crack propagation through the material [12]. Hygrothermal aging primarily damages the matrix, with this initial damage triggering other mechanisms such as fiber/matrix debonding and fiber breakage as part of the deterioration induced by aging [5]. 

The type of fiber and its composition also affect water absorption, with some fibers absorbing more moisture than others. There is currently a trend to replace synthetic fibers with natural fibers as reinforcements in polymer composites to reduce environmental impact. Similarly, replacing petroleum-based plastic with starch-based film has been an alternative for producing green composites [13,14].

The effects of water absorption in compounds reinforced by natural fibers are intensified. This happens mainly due to the poor interface of these fibers with the polymeric matrix as to hydrophilic characteristics by nature, which means that they have a natural affinity to absorb water, either by capillarity or because of the percentage of cellulose present in the fibers. A common problem is that the hydrophilic nature of plant fibers makes them incompatible with most polymeric resins, which are generally hydrophobic [8,15,16]. This way, a decrease in mechanical properties and an increase in moisture content is expected with the volume fraction of the fiber. This behavior is generally associated with the poor fiber/matrix interface. 

It is well-known that vegetable fibers have inferior mechanical properties compared to synthetic fibers [17,18,19]. Property losses have been observed in polymeric composites reinforced by plant fibers such as sisal [20], bamboo [21], flax [5], hemp [22,23], and jute [24]. The development of hybrid composites, where part of the fibers is synthetic and part is vegetable, is an alternative to overcome this issue [23,24,25,26,27]. Among the vegetable fibers, jute fibers excel in this application due to their widespread availability in numerous countries, cost-effectiveness, and possession of favorable mechanical properties [27,28]. However, the utilization of hybrid composites consisting exclusively of vegetable fibers or a combination of synthetic and vegetable fibers in high-performance applications should be conducted with prudence. Despite these factors, the use of vegetable fibers in hybrid composites can result in materials with suitable properties and be attractive in specific applications depending on project requirements [29,30,31]. 

Fibrous hybrid composites are materials that combine two or more distinct types of fibers to reinforce a particular matrix. They exhibit intermediate mechanical properties compared to the same composite reinforced by each fiber individually and offer various possibilities for fiber arrangements, including interlaminar, intralaminar, and mixed configurations [32,33,34,35,36]. Several factors play a role in influencing the properties of these hybrid composites, such as the mechanical properties and characteristics of the fibers, the length of the different fiber types, and the quality of the interfacial bonding between the fibers and the matrix [36].

Synergistic effects can be observed when the properties of hybrid composites are higher or lower than expected. The synergistic effect, also known as the hybrid effect, refers to the impact on the stress–strain response of mechanical loading in hybrid composites compared to non-hybrid composites [37]. The initial studies on the hybrid effect were reported between the early 1970s and 1980s [37,38,39,40,41,42]. It is defined in two different ways: one is based on the increase in the failure stress of the hybrid composite compared to non-hybrid fibers of low elongation [31,36], and the other is based on the rule of mixtures, used as a parameter to evaluate the deviation in mechanical behavior between hybrid and non-hybrid composites. However, in some cases, certain properties remain constant regardless of the amount of fibers added to the composite [35].

Although it is widely used, some caution must be exercised regarding the rule of mixtures. The rule of mixtures is not linear for all properties and, therefore, not suitable for estimating all mechanical properties. For instance, it is not recommended to use it for estimating the flexural strength of a composite [36]. The synergistic effect is also reported for hybrid composites with two or more resin systems or additional constituents, such as inserts, nanoparticles, and additives [43,44,45]. Another parameter that directly influences the mechanical properties of hybrid composites is the fiber stacking sequence. Moreover, there are situations where a property shows a negative hybrid effect for one characteristic while displaying a positive hybrid effect for another property [36]. For example, in carbon/glass hybrid composites, the ultimate stress at break displays a negative hybrid effect due to the positive hybrid effect on maximum strain [37].

The properties of jute composites can be enhanced by incorporating glass fiber as outer layers in the laminate, whereas jute should be used on the inside [46]. Several studies have explored the impact of incorporating glass fibers into composites reinforced with natural fibers [47,48,49,50,51,52,53,54,55,56]. In all cases, the consensus was that the inclusion of glass fibers led to a notable enhancement in the mechanical properties of these composites. However, a reduction in mechanical properties was correlated with moisture absorption [51]. Effects such as matrix cracks, delamination, fractures along the interface, resin particle loss, and fiber misalignment were observed due to the impact of moisture diffusion in hybrid composites [22]. When the glass fiber is used in the outer layer, lower water absorption is observed in comparison to other hybrid laminates with plant fabric on the outer layer [57].

The effect of water moisture on the mechanical performance of fiber-reinforced polymer matrix composites has been extensively studied in the literature [10,12,22,51,57,58,59,60]. However, there is still a need for further investigation regarding the sorption effect on the mechanical behavior of hybrid polyester/glass/jute composites, especially with different fiber stacking configurations, to fully understand its influence on material properties.

This study aims to assess how water absorption affects the tensile mechanical properties of composites at various temperatures. We investigate composites manufactured using different molding techniques and reinforcement stacking sequences. Two molding methods, compression molding and vacuum-assisted resin transfer molding (VARTM), were employed, producing laminates with five distinct fiber stacking sequences. Damage mechanisms were analyzed using SEM.

A previous study [61] conducted experimental and theoretical research on water absorption in these composites. The findings indicated that hybridization improved mechanical properties, with hybrid compounds showing intermediate results between glass fiber and jute fiber composites.

The novelty of our work lies in its unique approach. The existing literature lacks discussions on combinations and analyses used in evaluating hybrid compounds reinforced with plant and synthetic fibers after water immersion at varying temperatures, with diverse stacking sequences, and manufactured using two different processes. Hence, our study provides deeper insights into the mechanical behavior and damage mechanisms of dry and wet composite materials.

## 2. Materials and Methods

### 2.1. Materials

This study utilized orthophthalic unsaturated polyester resin 10316-10 produced for Reichhold, from Mogi das Cruzes, SP, Brazil (Table 1), catalyzed with MEKP BUTANOX M-50 supplied by IBEX Químicos e Compósitos Ltd.a., Recife, PE, Brazil. The reinforcements used were type E glass fiber fabrics with a gramature of 330 g/m^2^, as supplied by Redelease Ltd., Sorocaba, Brazil, and jute plain weave fabric with a gramature of 330 g/m^2^, manufactured by Cia. Têxtil Castanhal from Castanhal, PA, Brazil.

### 2.2. Manufacturing of Composites

**Figure 1:**
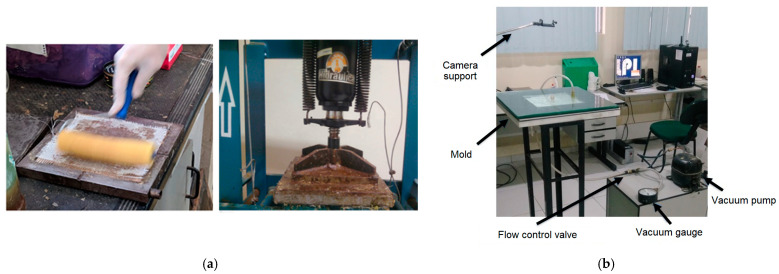
Manufacturing of composite plates: (**a**) compression molding; (**b**) VARTM.

The composites were manufactured using two different methods: compression molding (Figure 1a) and VARTM (Figure 1b).

**Figure 2:**
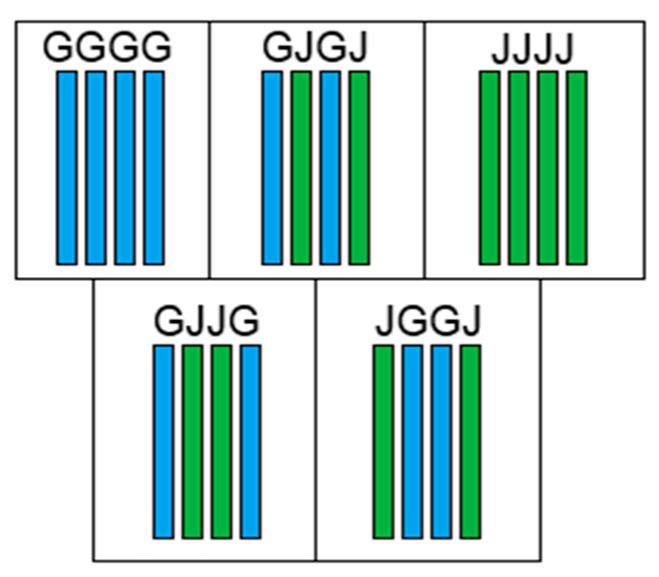
Stacking sequences; blue color represents glass fibers and green jute fibers.

Regardless of the method used for fabrication, each composite is composed of four layers of reinforcement, utilizing either glass fabric (G) or jute plain weave fabric (J) arranged in different stacking sequences (Figure 2): GGGG; JJJJ; GJGJ; JGGJ; GJJG. A comparison of fabrication methods (compression or VARTM) can be illustrated, for instance, by comparing GJJG-C and GJJG-R, where "C" denotes compression molding, and "R" denotes the VARTM process.

The laminates were manufactured with dimensions of 200 mm × 180 mm, both through compression molding and VARTM. In compression molding, the fabric layers were manually positioned in the metal mold, and lamination was carried out using a foam roller (Figure 1a). Compression molding was performed using hydraulic press produced for Marconi Equipamentos para Laboratórios Ltd.a, Piracicaba, Brasil, at room temperature with 9 Ton/24 h. In VARTM fabrication, the plates were produced in a mold with a glass base and vacuum bag (Figure 1b), employing a transverse flow front, two resin entry points, one exit point with a ¼” diameter, and a vacuum pressure of −0.3 bar. Post-curing of the compression molding and VARTM laminates was conducted in an air circulation oven at a temperature of 60 °C.

After fabrication, the plates were weighed, and the theoretical method [14] was employed to determine the volumetric fractions of the fibers (Equation (1)).

(1) Vf=wjρj+wgρgwjρj+wgρg+wmρm,

where the V*_f_* is the volumetric fraction of the fibers of the laminate; w*_j_*, *w_g_*, and *w_m_* are the masses of the jute fibers, glass fibers, and matrix, respectively; and ρ*_j_*, ρ*_g_*, and ρ*_m_* are the densities of the jute fiber, glass fiber and matrix, respectively. Thus, the percentage of fiber volume in the hybrid composites manufactured by compression molding was around 31%, while for glass fiber it was 39%, and for jute fiber it was 37%. With VARTM molding technique, the fiber content was 37%.

The laminates were cut on a CNC mill. The specimens obtained had dimensions of 100 mm × 13 mm × 3.5 mm for the tensile tests, adapting the recommendations of the ASTM D3039 standard [62].

In a previous work, both experimental and theoretical water sorption tests were conducted for these composites, determining the required exposure time for them to reach saturation [61]. These absorption curves served as a foundation for establishing the immersion duration in water for the tensile samples analyzed in this study.

### 2.3. Tensile Tests

The tensile tests were carried out in the MTS universal testing machine, model 810 with a 100 KN load cell, following the recommendations of the ASTM D3039 standard [62]. The tests were carried out at room temperature, with a displacement rate of 1 mm/min. In order to determine the influence of water absorption on the mechanical properties of the composites, the test was carried out with samples that were immersed in water for 696 h (saturation time) and dry samples, i.e., those not immersed in water. Water immersion temperatures were as follows: room temperature, 50 °C, and 70 °C. Tensile tests were conducted on samples immersed in water for 696 h at the three specified temperatures, as well as on samples exposed to 70 °C for times that led to an estimated 3% absorption (1.5% for glass fiber composites). The average results of five samples are reported for each set of samples manufactured.

### 2.4. Hybrid Effect Methodology

For the calculation of the hybrid effect, two methodologies were employed: one based on the rule of mixtures and another using a theoretical value for the strain at break of hybrid composites [63,64]. In the first methodology, the rule of mixtures was employed to establish a theoretical value for the stress at break of hybrid composites. Equation (2) is used when the volumetric fraction of the composite with higher strength (*V_fH_)* is lower than the critical volumetric fraction (*V_fcrit_*) [63]. Equation (3) is applied when the volumetric fraction of the composite with higher strength (*V_fH_*) is greater than the critical volumetric fraction *(V_fcrit_*) [63]. The critical volumetric fraction is a theoretical value obtained by equating Equations (2) and (3).

(2) $$\sigma_{htheoretical}=\left(1-V_{fm}\right)\left(\sigma_{H}V_{fLE}+\varepsilon_{LE}E_{H}V_{fH}\right);\;V_{fH}\leq V_{fcrit},$$

(3) $$\sigma_{htheoretical}=\left(1-V_{fm}\right)\left(\sigma_{H}V_{fH}\right);\;V_{fH}\geq V_{fcrit},$$

where *V_fm_* is the volume fraction of the matrix, *V_fLE_* is the relative volume fraction of fibers with lower strength, and E_H_ is the elastic modulus of the composite with higher strength. The calculation of the hybridization effect for stress is determined by taking the difference between the experimental stress value and the theoretical value (Equation (4)).

(4) $$\lambda_{stress}={\sigma_{hybrid}-\sigma }_{theoretical},$$

The hybrid effect can also be calculated from the theoretical value for the strain at break of hybrid composites (Equation (5)), and similar to stress, the calculation of the hybridization effect for strain is also performed by taking the difference between the experimental values for strain in hybrid composites and the theoretical value (Equation (6)) [63].

(5) $$\varepsilon_{htheoretical}={\varepsilon_{LE}V_{fLE}+\varepsilon }_{H}V_{fH},$$

(6) $$\lambda_{strain}={\varepsilon_{hybrid}-\varepsilon }_{htheoretical},$$

### 2.5. Scanning Eletron Microscope (SEM)

The fracture surfaces of tensile test samples were used to investigate sample morphology and to verify the damage mechanisms in the fracture region. The equipment used was a scanning electron microscope (SEM) Vega 3 microscope produced for Tescan, from Brno, Czech Republic. The fractured portions of the samples were cut and gold-coated uniformly over the surface for examination. The accelerating voltage used in this work was 20 kV. Only one sample each composition was tested.

## 3. Results and Discussion

### 3.1. Volumetric Fraction of Fibers

Table 2 presents the total and relative values of the weight and volumetric fractions of the fiber composites. For the theoretical calculation and results, fiber densities of 1.5 g/cm^3^ (jute) and 2.54 g/cm^3^ (glass) were utilized, as shown in Equation (1).

The volume fraction of fibers in the composites ranged from approximately 31% to 39%, with the hybrid composites manufactured by compression molding showing the lowest values. Hybrid composites fabricated using VARTM had an average volumetric fiber fraction of 37%. The difference in results between the manufacturing methods was anticipated, as VARTM promotes better fiber compaction during composite fabrication due to the use of a vacuum, in contrast to compression molding. The use of a vacuum, along with the resin flow front during the manufacturing process, enhances the effective removal of air, consequently decreasing the void content in the composites.

### 3.2. Tensile Tests Results

**Figure 3:**
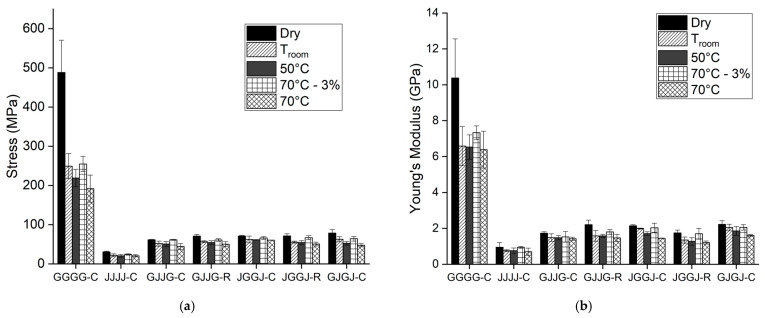
Mechanical properties of the composites under dry conditions and subjected to water sorption at different temperature conditions: (**a**) Stress at break; (**b**) Elastic modulus.

Figure 3 and Table 3 and Table 4 display the tensile test results of the samples for all analyzed conditions.

Figure 3 shows that the stress and elastic modulus results of the composites meet the expected outcomes for all analyzed conditions: dry samples, water immersion at room temperature, 50 °C, 70 °C for 696 h, and 70 °C with 3% absorption. The fiberglass composites exhibited the highest mechanical strength values, jute fiber composites displayed the lowest values, and the hybrid composites demonstrated intermediate values between fiberglass and jute fiber composites.

It was further noted that, despite different stacking sequences, the hybrid composites showed similar results for stress and modulus under all conditions and that the mechanical behavior of the hybrid composites was closer to that of jute fiber composites than fiberglass composites for all conditions. This was attributed to the higher volumetric fraction of jute fiber compared to fiberglass in the hybrid composites. The addition of glass fibers to jute fiber composites resulted in higher stress and modulus values, but the increase was marginal compared to fiberglass composites. The properties of hybrid composites depend on the relative volumetric fraction of each reinforcement, meaning that a higher relative volumetric fraction of a particular fiber will make the hybrid composite properties closer to those reinforced exclusively by that type of fiber [29,31].

Analyzing Table 2, it was found that the volumetric fraction of jute fibers was significantly higher than that of glass fibers in all hybrid composites. It is important to consider that the low adhesion between jute and glass fibers and the matrix created a low-quality interface, contributing to the low stress and modulus values exhibited by the hybrid composites. When moisture interacts with the fiber, it primarily penetrates through the cross-sectional area. This interaction between the hydrophilic fiber and the hydrophobic matrix causes the fiber to swell within the matrix. As a result, the bonding at the interface weakens, leading to dimensional instability, matrix cracking, and reduced mechanical properties of the composites [16].

Few differences were observed considering the manufacturing method, regardless of the test conditions (values were very close due to the identical composition of the composites; variables such as the stacking sequence and manufacturing method did not have a significant impact on the final results).

For the hybrid composites, statistical tests found no evidence to reject the null hypothesis, meaning that all hybrid composites showed equivalent results. This suggests that, although there are differences in water absorption at low rates for different stacking sequences [61], these differences do not reflect in mechanical behavior at saturation. Additionally, a reduction in stress at break values was observed for all composites exposed to moisture compared to dry composites. The more severe the conditions the composites were subjected to, the lower the observed stresses at break, indicating that the combination of moisture and temperature was highly detrimental to the materials. The higher the exposure temperature, the lower the observed stress at break, making it a determining factor in the mechanical behavior of composites, with 70 °C being the most severe condition adopted.

The choice of a fixed value for the amount of absorbed moisture by the samples was made to assess the effect of sorption on the mechanical properties of the composites, with equal water absorption for all samples over a different immersion time than the saturation time. The most severe condition (70 °C) was chosen, and the selection of the approximately 3% absorption value for the samples was based on the analysis of sorption graphs [61]. For fiberglass composites, tests were conducted with an absorption value of approximately 1.5%, as these composites reached saturation with about 3% water absorption.

For the maximum absorption condition of 3%, a considerable reduction in tensile strength was observed compared to the results of tests on dry composites, even for a shorter exposure period, highlighting the detrimental effect of moisture on the properties of this type of composite.

Dry composites exhibited better mechanical properties, followed by composites immersed at 70 °C with 3% absorption. This was followed by composites immersed in water for 696 h at room temperature, 50 °C, and 70 °C, respectively. These results were expected because higher moisture sorption leads to lower mechanical properties. The sorption kinetics increase with temperature, but the sorption level reached by the composites at the saturation time is similar for different conditions. Thus, after a certain time, composites immersed in water at room temperature will reach sorption values similar to those immersed at higher temperatures. Therefore, the higher the controlled temperature during immersion, the more rapidly the material will reach equilibrium sorption.

Fiberglass composites showed a trend of reduced properties with increasing temperature during water sorption tests until saturation. Only the fiberglass composite immersed at 70 °C with 1.5% absorption showed no variation in properties, considering standard deviations. Jute fiber composites maintained the trend of mechanical property loss with water sorption for all conditions. This behavior can be attributed to the degradation of the polyester matrix when subjected to water immersion. Thus, it was possible to observe that, except for these specific conditions, the temperature factor did not show significant variation in material stress.

Through the fiber stacking sequence, it was observed that there was a tendency for an increase in mechanical properties when fiberglass fibers were positioned in the center of the composite. Placing fiberglass fibers in the center of the composite improved the quality of the interface between the resin and these fibers. The same behavior was observed for the modulus values of all materials.

Few differences were observed between composites immersed in water for 696 h because, at this immersion time, the composites had already reached equilibrium sorption for all immersion temperatures. Larger differences were observed between dry composites compared to the results of tests under other conditions, highlighting the effect of moisture on mechanical properties.

Considering all the studied conditions, it was possible to conclude that, although some differences between the types of hybrid composites were observed under certain conditions, overall, the various fiber arrangements in the composites had little influence on their mechanical properties.

### 3.3. Hybrid Effect

**Figure 4:**
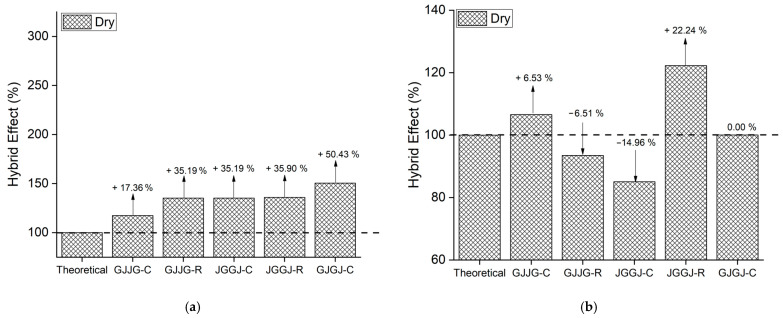
Hybrid effect of dry composites: (**a**) stress; (**b**) strain.

**Figure 5:**
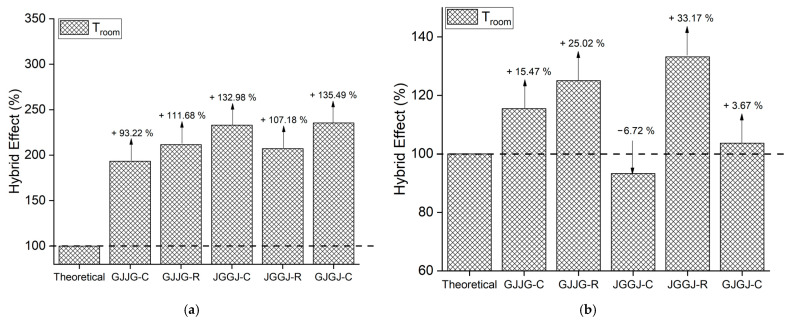
Hybrid effect of dry composites at room temperature: (**a**) stress at break; (**b**) strain at break.

**Figure 6:**
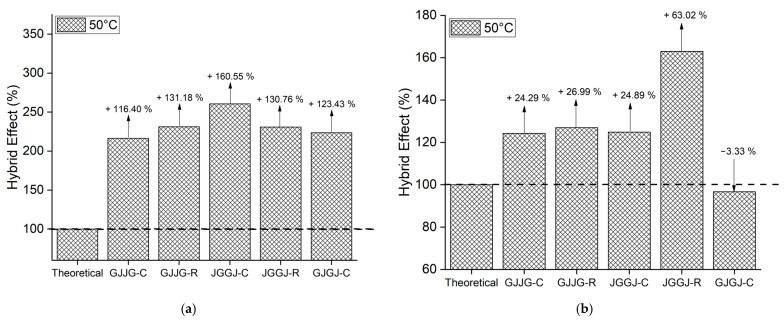
Hybrid effect of wet composites at 50 °C: (**a**) stress at break; (**b**) strain at break.

**Figure 7:**
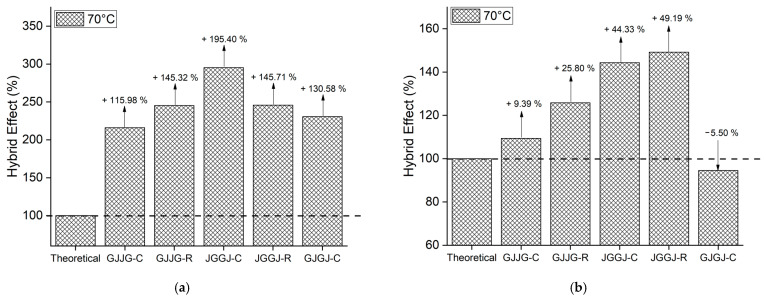
Hybrid effect of wet composites at 70 °C: (**a**) stress at break; (**b**) strain at break.

**Figure 8:**
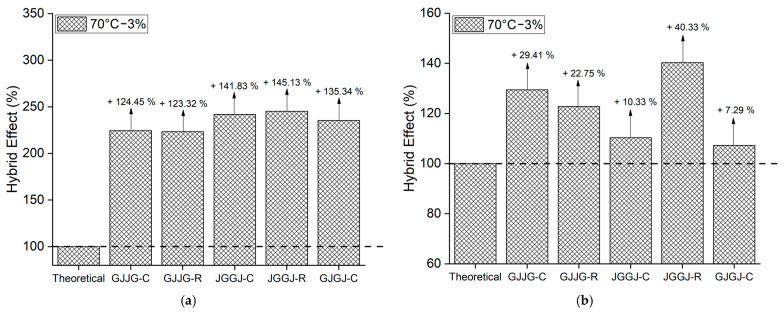
Hybrid effect of wet composites at 70 °C with 3% water absorption: (**a**) stress at break; (**b**) strain at break.

When the strength values are higher than the theoretical values, this indicates that the composite in question exhibited a positive hybrid effect, just as strength values lower than the theoretical values signify a negative hybrid effect. The hybrid effect of the composites is depicted in Figure 4, Figure 5, Figure 6, Figure 7 and Figure 8 for the dry (Figure 4), as well as room-temperature (Figure 5), 50 °C (Figure 6), 70 °C (Figure 7), and 70 °C–3% (Figure 8) conditions.

In Figure 4a, a positive hybridization effect for stress at break was observed for all composites, where the composite GJJG-C showed the lowest value (+17.36%), and the composite GJGJ-C exhibited the highest value (+50.43%). The other composites presented intermediate values: GJJG-R (+35.19%), JGGJ-C (+35.19%), and JGGJ-R (+35.90%). In Figure 3b, the hybridization effect for strain at break is negative for the composites GJJG-R (−6.51%) and JGGJ-C (−14.96%), both showing values below the expected value for these composites. The composites GJJG-C (+6.53%) and JGGJ-R (+22.24%) showed a positive hybridization effect for strain at break, while the strain at break of the composite GJGJ-C was equivalent to the theoretical deformation, showing neither a positive nor negative hybridization effect.

The variation in the hybridization effect in Figure 3b may be associated with the processing of the JGGJ composites, as the JGGJ-C composite exhibited a negative hybridization effect, while the JGGJ-R composite showed a positive hybridization effect. One consideration is that when fibers with lower strength have a higher volumetric fraction than fibers with higher strength, hybrid composites fail when the tensile strain reaches a value close to the failure strain of composites reinforced with the lower-strength fiber [63] (in our case, jute). However, if the higher-strength fiber has a greater volumetric fraction, the lower-strength fibers fail at the beginning, but the hybrid composites still maintain their integrity until the failure of the higher-strength fibers due to their greater failure strain.

Upon analyzing Figure 5a, Figure 6a, Figure 7a and Figure 8a, it is evident that all composites exhibit a positive hybrid effect for stress during exposure to room temperature, 50 °C, and 70 °C for 696 h, as well as 70 °C–3%. The values for the hybridization effect observed for stress were considerably higher than those observed in dry composites. The reason for this difference in values occurred due to the presence of moisture and/or temperature, causing a significant reduction in the stress of the fiberglass composites compared to the dry composites, which were used as a reference to calculate the theoretical value. Equation (6) used for calculating the theoretical stress takes into account the stress value of the composite with higher strength (fiberglass composite). In Figure 3, it can be observed that fiberglass composites exhibited much lower average stress when subjected to water sorption/temperature, directly affecting the values obtained for the hybridization effect studied here.

As for strain (Figure 5b, Figure 6b, Figure 7b and Figure 8b), the hybridization effect presented results that were similar to dry composites, although a slight increase in values was observed for all degradation conditions. An increase in the value of the hybridization effect was expected since Equation (5) uses the strain values of fiberglass composites and jute fiber composites to determine the theoretical strain of hybrid composites through the rule of mixtures. As there was a decrease in the strain values of fiberglass composites and jute fiber composites exposed to moisture compared to dry composites, these increases in the hybridization effect are justified. An increase in the strain of composites exposed to moisture is also expected due to the plasticization of the matrix caused by water absorption.

### 3.4. Damage Mechanism of Dry and Wet Composites

**Figure 9:**
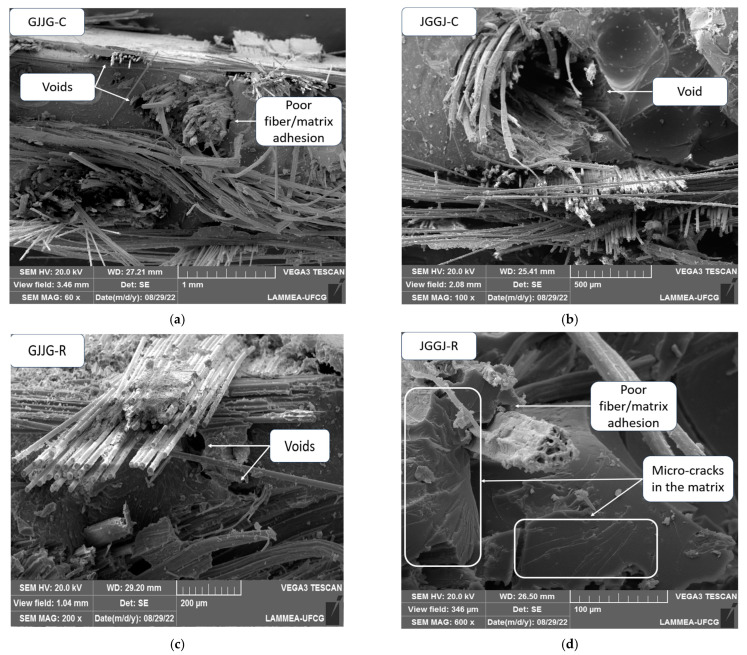
SEM images of dry hybrid composites with different stacking sequences: (**a**) composite GJJG-C; (**b**) JGGJ-C; (**c**) GJJG-R; (**d**) JGGJ-R.

SEM images of the fracture surface of the composites were obtained for both dry and water-immersed samples at 70 °C for the hybrid composites. Figure 9 displays the SEM images of the fracture surface of the dry composites.

Figure 9 reveals various failure mechanisms in the composites subjected to tensile testing. Fiber breakage, matrix fracture, fiber debonding, and fiber pull-out can be observed after the tensile test in all composites. It was also possible to clearly identify the different fibers and the matrix in the composites, and even after fracture, the glass fibers showed a certain regularity of arrangement, unlike the jute fibers, which appeared more irregular (Figure 9b,c). The difference in fiber arrangement within the composite has a direct influence on their mechanical behavior [65].

In Figure 9a–c, it is possible to identify voids in the composites, as well as fractures of both longitudinal glass and jute fibers along the length of the specimens. These voids were caused by fiber pull-out during tensile tests and were observed in all hybrid composites. In Figure 9b, complete removal of jute fiber bundles can be seen. In Figure 9a,d, debonding between jute fibers and the polymeric matrix is also observed, along with poor wetting of the fibers by the resin, resulting in a low-quality interface due to little or no adhesion between the phases. Fiber pull-out and debonding between the fibers and the matrix are the main factors responsible for the reduction in tensile and elastic modulus values [2,25,51,52,66]. One of the functions of a composite matrix is to transfer the load to the fibers through interfacial shear stress, so the fracture behavior also depends on the interfacial strength [25].

In Figure 9d, micro-cracks in the form of streaks that formed in the polymeric matrix can also be observed. One of the main failure mechanisms observed in hybrid composites was caused by the propagation of micro-cracks in the matrix [5]. The cracks propagate easily through the matrix, indicating that little resistance is offered, as evidenced by the poor interfacial bonding observed from the fracture [65]. In hybrid composites, when the load is applied, cracks in the matrix occur before the final failure [67].

**Figure 10:**
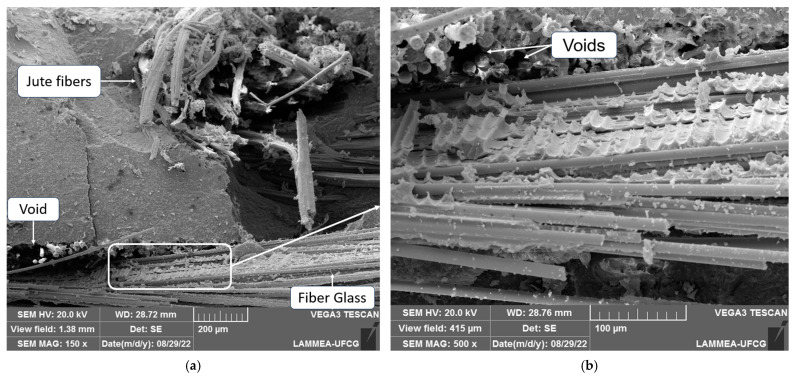
Low adhesion of the glass fibers in the dry composites: (**a**) 150× zoom; (**b**) 500× zoom.

Figure 10 shows that, in addition to the jute fibers, the glass fibers also exhibited poor adhesion with the matrix, where it was possible to identify the glass fibers, jute fibers, and the matrix phase. Voids caused by the pull-out of glass fibers at that location were observed. In Figure 10a, a highlighted region is magnified, as illustrated in Figure 10b, where it is evident that even the interface between the glass fibers and the resin shows low quality, with regions where the fibers were pulled out arranged in the longitudinal direction of the sample, and poor wetting of the glass fibers by the resin also observed.

**Figure 11:**
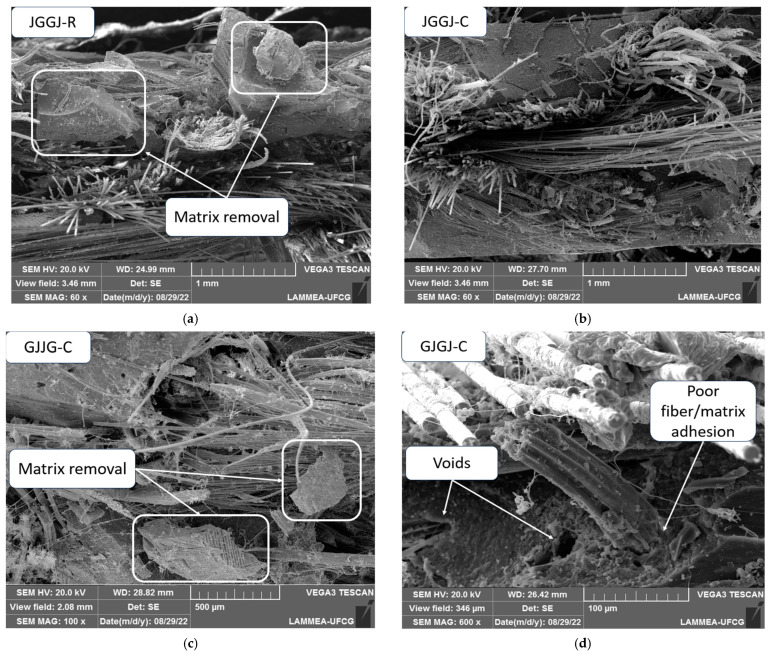
SEM images of wet hybrid composites with different stacking sequences at 70 °C: (**a**) composite JGGJ-R; (**b**) JGGJ-C; (**c**) GJJG-C; (**d**) GJJG-R.

Figure 11 illustrates SEM images of the fracture surface of the composites subjected to water sorption at 70 °C. It can be observed that the issues in the dry composites related to fiber pull-out, voids, fiber fracture, and low adhesion between fibers and the matrix were also observed in these composites. In addition to the aforementioned problems, removal and fragmentation of the matrix were also observed due to its degradation caused by moisture (Figure 11a–c). Matrix degradation on the fracture surface was also observed (Figure 11d).

**Figure 12:**
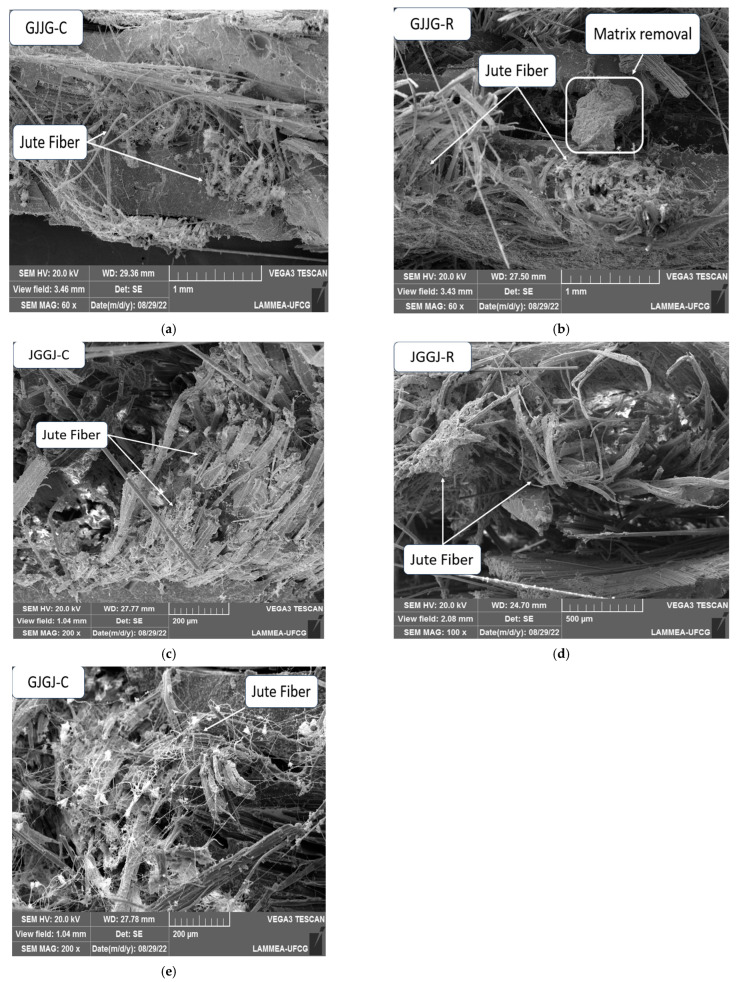
SEM images of the degradation mode of wet hybrid composites with different stacking sequences at 70 °C: (**a**) GJJG-C; (**b**) GJJG-R; (**c**) JGGJ-C; (**d**) JGGJ-R; (**e**) GJGJ-C.

In Figure 11c, the degradation of jute fibers can be further observed, as if they had “unraveled”, generating a tangle of jute fibers with glass fibers and fragmented matrix particles. Based on this observation, Figure 12 was generated, showing a microscopy image of each hybrid composite sample that was exposed to moisture to assess whether this result was repeated for all composites.

The analysis of Figure 12 shows that this result was repeated for all types of composites analyzed, regardless of the manufacturing method or arrangement of jute fibers. This phenomenon can also be referred to as a microfibril explosion due to the action of moisture [5]. Hygrothermal aging, specifically at higher temperatures, does not induce other damage mechanisms to the composite; it merely accelerates degradation due to plasticization and reorientation of microfibrils in jute fibers [5]. Since the loss of mass in jute fibers only occurs at temperatures around 150 °C, we can currently consider that the microfibril explosion was caused by excess moisture absorbed by the jute fibers and not by an effect of temperature. In future work, it would be interesting to verify whether the same behavior of jute fibers occurs in samples subjected to other conditions studied in this work or only at 70 °C.

Observations in Figure 11 and Figure 12 allow us to conclude that degradation, removal, and fragmentation of the matrix, as well as microfibrillation in jute fibers, were the main mechanisms (observed in SEM) responsible for the lower mechanical performance of composites subjected to water sorption at 70 °C compared to dry composites. This is because failure mechanisms such as fiber pull-out, voids, fiber fracture, and low adhesion between fibers and the matrix were observed in composites in both conditions. Although composite swelling due to moisture absorption is considered a factor of the reduction in mechanical properties due to interfacial degradation of phases, this was not observed in scanning electron microscopy.

## 4. Conclusions

This study investigated the effect of water absorption on the mechanical behavior and damage mechanisms in hybrid polyester/glass fiber/jute fiber composites with various stacking sequences. It was found that the addition of glass fiber enhanced the mechanical properties of jute fiber composites. Glass fiber composites consistently exhibited the highest mechanical properties, while jute fiber composites demonstrated the lowest mechanical properties, with hybrid composites falling between these extremes. Interestingly, hybrid composites displayed behavior more closely resembling that of jute fiber composites rather than glass fiber composites. Overall, the different fiber arrangements showed minimal influence on mechanical properties across the various conditions studied.

Furthermore, this study observed few disparities in mechanical properties between composites fabricated via compression molding and VARTM methods. Dry composites performed the best, followed by those aged at 70 °C with approximately 3% water absorption. Subsequently, composites immersed in water at room temperature for 696 h, in water at 50 °C for 696 h, and in water at 70 °C for 696 h showed progressively poorer mechanical properties. These results were expected and were attributed to the varying degrees of water absorption by the systems.

Moreover, positive hybridization effects on stress were noted across all conditions examined. However, certain composites exhibited negative effects on deformation under specific conditions. SEM images of fractured samples revealed a weak interface between the matrix and fibers, showcasing different fiber and matrix phases, fiber pull-out, voids, matrix cracks, moisture-induced matrix removal, and poor adhesion between fibers and the matrix. These findings offer valuable insights into the mechanical behavior and structural integrity of composite materials under different environmental conditions.

## Figures and Tables

**Table 1:** Characteristics of resin—Reichhold guideline.

Characteristics	Values
Brookfield viscosity at 25 °C sp3: 60 rpm (CP)	250–350
Thixotropy index	1.30–2.10
Solid content—Reichhold method (%)	55–63
Density at 25 °C (g/cm^3^)	1.07–1.11
Acidity index (mgKOH/g)	30 maximum
Exothermic curve at 25 °C	
- Gel time (min) - Single interval (min) - Maximum temperature (°C)	5–7 8–14 140–180
Post-cure	60 °C

**Table 2:** Total and relative fiber weight and volume fraction of manufactured composites.

Composites	Glass Fiber Weight Fraction (%)	Jute Fiber Weight Fraction (%)	Total Fiber Weight Fraction of Composites (%)	Fiber Volume Fraction of Composites (%)	Total Fiber Volume Fraction of Jute (%)
GGGG-C ^1^	59.70 ± 3.27	0	59.70 ± 3.27	39.35 ± 3.21	0
JJJJ-C	0	44.10 ± 2.10	44.10 ± 2.10	36.87 ± 1.97	36.87 ± 1.97
GJGJ-C	15.23 ± 0.43	26.10 ± 0.74	41.33 ± 1.17	30.68 ± 1.02	24.77 ± 0.90
GJJG-C	15.23 ± 1.06	26.11 ± 1.82	41.34 ± 2.89	30.72 ± 2.55	24.81 ± 2.24
GJJG-R ^1^	17.77 ± 1.77	30.46 ± 3.04	48.23 ± 4.82	36.99 ± 4.48	30.42 ± 4.06
JGGJ-C	15.28 ± 1.16	26.19 ± 2.00	41.47 ± 3.16	30.83 ± 2.76	24.91 ± 2.42
JGGJ-R	18.15 ± 1.18	31.12 ± 2.02	49.28 ± 3.20	37.94 ± 3.00	31.27 ± 2.73

^1^ C and R indicate the manufacturing method: compression molding (C) and VARTM (R).

**Table 3:** Fracture stress of composites for all conditions.

Fracture Stress (MPa)					
Composites	Dry	Room	50 °C	70 °C	70 °C–3%
GGGG-C	488.22 ± 82.21	249.29 ± 32.28	219.01 ± 22.36	191.78 ± 34.61	254.60 ± 19.43
JJJJ-C	30.36 ± 1.87	21.89 ± 3.67	20.65 ± 3.28	20.53 ± 2.74	23.83 ± 1.66
GJJG-C	61.39 ± 0.88	51.61 ± 6.46	50.78 ± 6.29	44.38 ± 7.44	61.23 ± 1.21
GJJG-R	70.72 ± 0.53	56.54 ± 2.67	54.25 ± 4.80	50.41 ± 6.20	60.92 ± 3.44
JGGJ-C	70.72 ± 2.04	62.23 ± 8.47	61.14 ± 0.72	60.70 ± 0.18	65.97 ± 3.39
JGGJ-R	71.09 ± 5.73	55.34 ± 2.64	54.15 ± 4.84	50.49 ± 4.92	66.87 ± 5.23
GJGJ-C	78.69 ± 8.76	62.90 ± 6.67	52.43 ± 4.37	47.38 ± 4.26	64.20 ± 6.03

**Table 4:** Elastic modulus of composites for all conditions.

Elastic Modulus (GPa)					
Composites	Dry	Room	50 °C	70 °C	70 °C–3%
GGGG-C	10.38 ± 2.18	6.59 ± 1.08	6.53 ± 0.68	6.39 ± 1.03	7.33 ± 0.38
JJJJ-C	0.95 ± 0.26	0.76 ± 0.05	0.76 ± 0.15	0.70 ± 0.20	0.94 ± 0.05
GJJG-C	1.73 ± 0.08	1.49 ± 0.21	1.48 ± 0.12	1.42 ± 0.09	1.53 ± 0.30
GJJG-R	2.21 ± 0.25	1.58 ± 0.30	1.58 ± 0.09	1.47 ± 0.20	1.80 ± 0.14
JGGJ-C	2.14 ± 0.07	1.99 ± 0.03	1.71 ± 0.10	1.45 ± 0.01	2.03 ± 0.26
JGGJ-R	1.75 ± 0.16	1.35 ± 0.17	1.28 ± 0.21	1.21 ± 0.09	1.70 ± 0.30
GJGJ-C	2.22 ± 0.21	2.05 ± 0.18	1.86 ± 0.25	1.61 ± 0.05	2.06 ± 0.15

## Data Availability

Data are contained within the article.
